# A report of *Cylindrospermopsis raciborskii* and other cyanobacteria in the water reservoirs of power plants in Ukraine

**DOI:** 10.1007/s11356-018-2010-6

**Published:** 2018-04-21

**Authors:** Piotr Rzymski, Oksana Horyn, Agnieszka Budzyńska, Tomasz Jurczak, Mikołaj Kokociński, Przemysław Niedzielski, Piotr Klimaszyk, Halina Falfushynska

**Affiliations:** 1 Department of Environmental Medicine, Poznan University of Medical Sciences, Poznań, Poland; 2 Research Laboratory of Comparative Biochemistry and Molecular Biology, Ternopil National Pedagogical University, Ternopil, Ukraine; 3 Department of Water Protection, Adam Mickiewicz University, Poznań, Poland; 4 Department of Applied Ecology, Faculty of Biology and Environmental Protection, University of Łódź, Łódź, Poland; 5 Department of Hydrobiology, Adam Mickiewicz University, Poznań, Poland; 6 Department of Analytical Chemistry, Adam Mickiewicz University, Poznań, Poland

**Keywords:** *Cylindrospermopsis raciborskii*, Ukraine, Cyanotoxins, Cyanobacteria

## Abstract

The occurrence of cyanobacteria in freshwaters attracts much attention due to its associated health threats and ecological implications. Yet data on the composition of cyanobacteria taxa and toxigenicity in some regions is still scarce. Here, we explored the occurrence of cyanobacteria and cyanotoxins in three locations in Ukraine (reservoir for Kasperivtsi Hydrothermal Power Plant and outflowing River Seret, and cooling pond of Khmelnytsky Atomic Power Plant) in summer 2017. Cyanobacteria were a dominant fraction at all stations. A number of potent-toxin producers were identified including *Cylindrospermopsis raciborskii*, *Aphanizomenon gracile*, *Dolichospermum flos-aquae*, and *Planktothrix agardhii*. Screening for the presence of dissolved and particulate content of microcystins (-LR, -YR, and -RR), cylindrospermopsin, and anatoxin-a yielded negative results. The studied waters displayed no toxicity in human platelets in vitro. Further toxicological and ecological studies are necessary to evaluate the potential presence of cyanotoxin producers in Ukraine.

## Introduction

Cyanobacteria from the Chroococcales, Nostocales, and Oscillatoriales orders are subject to ongoing interest due to their nearly global distribution, potent production of various toxins, wide range of ecological adaptations, and ability to expand to new habitats. Their occurrence in freshwaters is promoted by cultural eutrophication but can also be driven by climate changes. As predicted, the incidence of harmful cyanobacteria blooms in various regions will increase advocating a need to monitor toxigenic species in various regions (Paerl and Paul 2011; O’Neil et al. 2012).

Particular attention is being paid to species that are recognized as potent producers of cyclic hepatopeptides microcystins (MCs) that include *Microcystis* *aeruginosa* (Kütz.) Kützing, *Planktothrix agardhii* (Gomont) Anagnostidis & Komárek, and members of *Nostoc*, *Anabaena*/*Dolichospermum*, and *Anabaenopsis* (Bernard et al. 2017), as well as potent producers of the neurotoxic (homo)anatoxin-a (ANA-A) alkaloid that encompass species belonging to the *Anabaena*, *Aphanizomenon*, and *Dolichospermum* genera. Moreover, the occurrence of cytotoxic cylindrospermopsin (CYN) in European freshwater has been increasingly reported with strains of *Aphanizomenon gracile* (Lemm.) Lemmermann, *A*. *klebahnii* Elenkin ex Pechar, *A*. *flos-aquae* Ralfs ex Bornet et Flahault, *A*. *ovalisporum* Forti, and *Oscillatoria* sp. recognized as potent producers (Rzymski and Poniedziałek 2014).

In turn, *Cylindrospermopsis raciborskii* (Woloszyńska) Seenayya et Subba Raju, known as the main producer of CYN in sub-tropical and tropical areas, has never been documented to produce any known cyanotoxin in Europe although numerous studies have already demonstrated the toxicity of their exudates using in vivo and in vitro experimental models (Acs et al. 2013; Smutná et al. 2016; Rzymski et al. 2017a). Reported first in Lake Kastoria in Greece (Skuja 1937), it has systematically been found throughout the continent, although was rarely observed to form blooms (Budzyńska and Gołdyn 2017). With recent reports of its common occurrence in western parts of Poland, in a single lake in Lithuania (Kokocinski et al. 2017; Rzymski et al. 2017b) and Lake Nero in the Yaroslavl Region of Russia (Babanazarova et al. 2015), it appears that *C*. *raciborskii* continues to spread. Its distribution pattern and threats associated with its expansion in Eastern Europe are, however, still not sufficiently explored.

Here, we report the occurrence of *C*. *raciborskii* and other cyanobacteria belonging to the Chroococcales, Nostocales, and Oscillatoriales orders in three locations in Ukraine (water reservoir for Kasperivtsi Hydrothermal Power Plant and outflowing River Seret and cooling pond of Khmelnytsky Atomic Power Plant) along with analyses of cyanotoxins (CYN, MCs, and ANA-A), physicochemical parameters, and in vitro toxicity of water.

## Material and methods

### Study area and water sampling

**Fig. 1:**
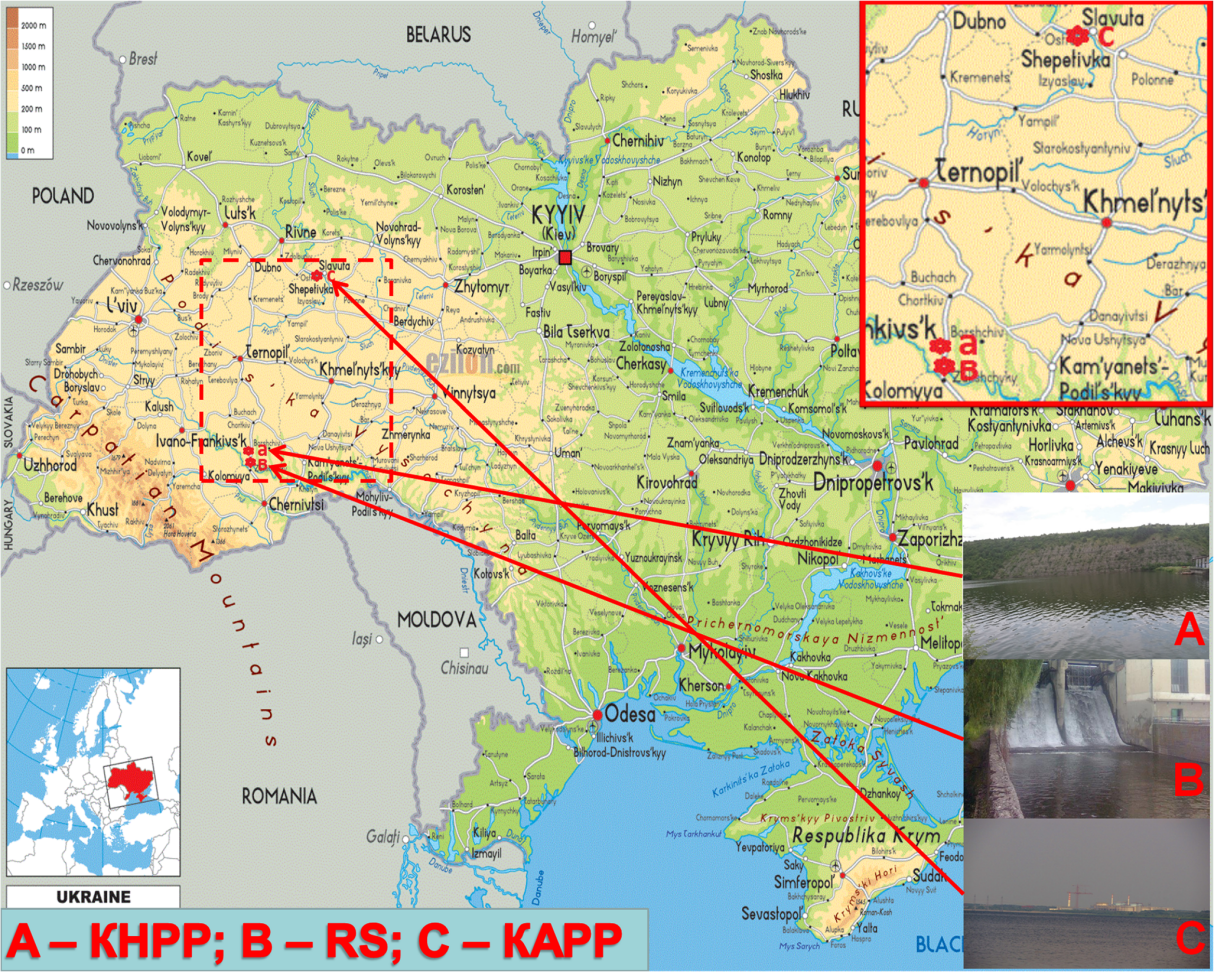
Localization of the sampling sites in Western Ukraine. Sites: A (KHPP)—Kasperivtsi water reservoir before dam; B (RS)—River Seret outflowing from Kasperivtsi water reservoir; C (KAPP)—cooling pond of Khmelnytsky Atomic Power Plant

The samplings were carried out during August and September 2017 according to Environmental Protection Agency recommendations (Surface Water Sampling 2013). Surface water samples were collected at three sites (KHPP, RS, and KAPP) located in the Galicia-Volyn area, Western Ukraine (Fig. 1), transferred to propylene bottles, and transported to the laboratory for determination of chemical parameters, phytoplankton analyses, and toxicological studies. The Kasperivtsi hydroelectric power plant is situated near the Seret riverbed (48° 40′ N, 25° 51′ E). It is a small power plant with the installed capacity of 7.5 MW. Water discharged from the Kasperivtsi dammed water reservoir (KHPP site) flows through a turbine directly into the River Seret (RS site). The Kasperivtsi water reservoir (surface area 2.86 km^2^ and length 12 km) is a recreation area and the part of territory of National Nature Park “Dnister Canyon” where no industrial contamination is expected. The KAPP site, with a consistently higher water temperature, is located on the bank of the cooling pond of Khmelnytsky Atomic Power Plant (APP) in Netishyn (in a forestry area on the tributary of the River Goryn, 50° 21′ N, 26° 38′ E). There is no connection via water between these sites; the distance between the KHPP/RS and KAPP sites is about 300 km.

### Phytoplankton analyses

The samples were preserved with Lugol solution and then analyzed within a month from sampling. Qualitative and quantitative phytoplankton analysis was performed in a Sedgwick-Rafter chamber of 0.5 mL volume, with the use of a light microscope Olympus BX60, according to standard methods (Wetzel and Likens 1991). Cyanobacterial and eukaryotic phytoplankton species were identified on the basis of the microscopic analysis of their morphological features, according to modern keys based on the polyphasic taxonomical approach (Komárek and Anagnostidis 1998; Komárek and Anagnostidis 2005; Komárek 2013).

### Physicochemical water analyses

Water parameters were measured by routine analytical tests. pH was measured using an MI 150 tester with a combination electrode. In the field, water for chemical analyses was placed without preservation into polyethylene flasks and transported in coolers to the laboratory. The following water parameters were analyzed: nitrogen forms (N-NH_4_^+^ using the colorimetric method with Nessler reagent, N-NO_2_^−^ colorimetric with Griess reagent, N-NO_3_^−^ colorimetric with sodium salicylate) and total reactive phosphorus (TRP, using the molybdate method) (APHA 1995). Phenol concentration was evaluated using the 4-*aminoantipyrine* method (Dannis 1951). The chloride ion concentration was analyzed using the trimetric with silver nitrate method (EPA norm 9253 1994). S*ulfate* concentration in *water* samples was determined by indirect EDTA titration (Belle-Oudry 2008).

Concentrations of calcium (Ca), magnesium (Mg), sodium (Na), potassium (K), manganese (Mn), iron (Fe), aluminum (Al), cadmium (Cd), cobalt (Co), chromium (Cr), copper (Cu), mercury (Hg), nickel (Ni), lead (Pb), and zinc (Zn) were quantified with the inductively coupled plasma optical emission spectrometer Agilent 5100 ICP-OES (Agilent, USA) after acidification of samples with nitric acid (Sigma-Aldrich, Germany) with conditions, wavelengths, and limit of detection (LOD) as described previously (Rzymski et al. 2017c). Calibration was performed using standard analytical solutions (Merck, Germany).

### Cyanotoxin analyses

Water samples in volume from 200 to 500 mL were filtered through GF/C filters (Whatman, UK) to separate cyanobacterial cells from water and to determine dissolved and particulate concentration of cyanotoxins (microcystins (MCs), anatoxin-a (ATX), and cylindrospermopsin (CYN)) using the HPLC-DAD method.

MCs in the suspended material were extracted in 75% aqueous methanol and CYN and ATX were extracted in methanol. The samples were sonicated for 30 s in a Misonix (Farmingdale, NY, USA) ultrasonicator equipped with an ultrasonic probe (100 W, diameter 19 mm with “spike”) and the liquid processor XL. The extracts were then centrifuged twice at 11000×*g* for 10 min at 4 °C in an Eppendorf 5804 centrifuge (Hamburg, Germany). The supernatants were collected and evaporated in a SC110A Speedvac^®^ Plus, ThermoSavant (Holbrook, NY, USA).

For dissolved MCs and ATX, samples of filtered water were concentrated on Baker (Deventer, Netherlands) C_18_ solid phase extraction (SPE) cartridges (sorbent mass 500 mg), conditioned earlier by 10 mL of methanol and water. Cyanotoxins were eluted from the C_18_ cartridges by 3 mL of 90% aqueous methanol containing 0.1% trifluoroacetic acid (TFA). The eluates were evaporated to dryness in a SC110A Speedvac^®^ Plus, ThermoSavant (Holbrook, NY, USA). For dissolved CYN polygraphite carbon (PGC), solid phase extraction cartridges (sorbent mass 200 mg) and C_18_ solid phase extraction cartridges were used in series (C_18_ before PGC). For conditioning of the combined system, 10 mL of methanol containing 0.1% (*v*/*v*) TFA followed by 10 mL of water was used. CYN were eluted by 3 mL of 0.1% (*v*/*v*) TFA in methanol from PGC cartridges and then evaporated to dryness.

Before HPLC analysis, the samples were redissolved in 75% aqueous methanol for MC analyses and in water for CYN and ATX analyses and filtered through a Gelman GHP Acrodisc 13-mm syringe filter with a 0.45-μm GHP membrane and minispike outlet (East Hills, NY, USA). Chromatographic separation was performed using an Agilent (Waldbronn, Germany) 1100 series HPLC system consisting of a degasser, a quaternary pump, a column compartment thermostat set at 40 °C, and a diode array detector operated at 200–300 nm on a Merck (Darmstadt, Germany) Purospher STAR RP-18e column (55 mm × 4 mm I.D. with 3-μm particles) protected by a 4 mm × 4 mm guard column. The mobile phase consisted of water (solvent A) and acetonitrile (solvent B), both containing 0.05% trifluoroacetic acid. The flow rate was 1.0 mL/min with the following linear gradient program: 0 min, 1% B; 5 min, 7% B; 5.1 min, 70% B; 7 min, 70% B; 7.1 min, 1% B; stop time, 12 min for CYN and ATX analyses. The injection volume was 20 μL. CYN in the samples was identified by comparing the retention time and UV spectrum (200–300 nm) with an absorption maximum at 262 nm for CYN and at 227 nm for ATX. The flow rate for MC analyses was 1.0 mL min^−1^ with the following linear gradient program: 0 min, 25% B; 5 min, 70% B; 6 min, 70% B; 6.1 min, 25% B; stop time, 9 min. The injection volume was 20 μL. The contents of MC-LR, MC-YR, and MC-RR in the samples were analyzed by comparing the retention time and UV spectrum (200–300 nm with an absorption maximum at 238 nm).

### In vitro toxicity assessment

The cytotoxicity of sampled water was assessed in an in vitro experimental model employing platelet-rich plasma (PRP) and assessing lactate dehydrogenase (LDH) leakage. This model was selected as cyanobacterial compounds can exhibit toxicity in platelets (Selheim et al. 2005). PRP was isolated by centrifugation (200×*g*, 12 min) from blood collected from five healthy donors at the Regional Centre of Blood and Blood Treatment in Poznan, Poland, according to accepted safeguard standards and legal requirements in Poland. One milliliter of PRP was incubated with 100 μL of filtered water samples for 1 h at 37 °C in darkness. Negative and positive controls were constituted of PRP incubated with phosphate-buffered saline and 10 μM of tert-butyl hydroperoxide (tBHP), respectively. The cytotoxicity was evaluated using Cytotoxicity Detection LDH Kit (Sigma-Aldrich, Germany) according to the manufacturer’s instructions. Briefly, following the incubation, all samples were centrifuged for 10 min at 1000 rpm and supernatants (100 μL) were transferred to a 96-well flat bottom microplate and mixed with a 100-μL reaction cocktail containing iodonitrotetrazolium chloride, sodium lactate, and diaphorase/NAD+ mixture. After incubation (30 min, 25 °C, darkness), the absorbance of each sample was read at 492 nm, and the cytotoxicity of the lake water sample was calculated according to the equation:

$$ Cytotoxicity\ \left[\%\right]=\frac{\mathrm{Absorbance}\ \left(\mathrm{sample}\right)-\mathrm{Absorbance}\ \left(\mathrm{control}\right)}{\mathrm{Absorbance}\ \left(\mathrm{high}\ \mathrm{control}\right)-\mathrm{Absorbance}\ \left(\mathrm{control}\right)}\times 100 $$

where control is a non-exposed sample and high control represents a non-exposed sample mixed with RIPA lysis buffer to evaluate the maximum releasable LDH activity for each sample.

## Results and discussion

**Fig. 2:**
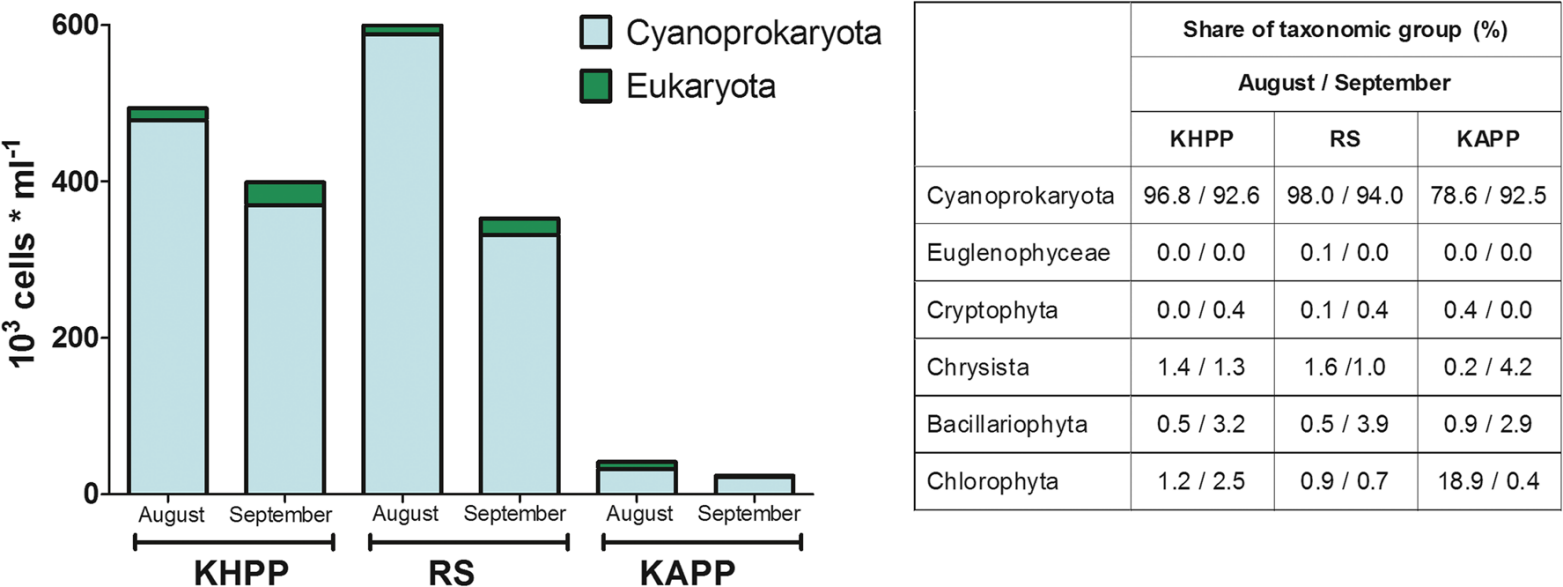
The abundance of cyanobacteria at three studied sites in August and September 2017

**Fig. 3:**
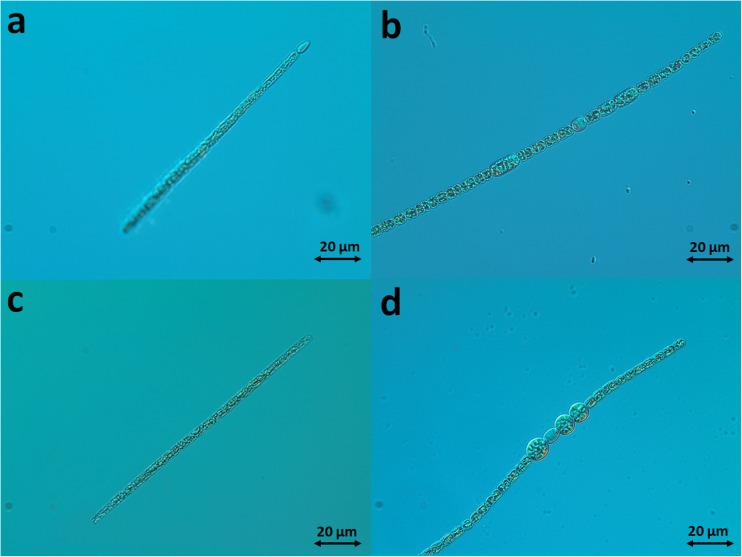
Cyanobacteria identified at KHPP and RS sites. **a** *Cylindrospermopsis raciborskii*, **b** *Aphanizomenon gracile*, **c** *Planktothrix agardhii*, and **d** *Sphaerospermopsis aphanizomenoides*

The physicochemical parameters of water during the sampling period are summarized in Table 1. The concentrations of toxic elements were low, indicating no industrial pollution although one should note that metal levels in water can be a subject to seasonal variations (Rzymski et al. 2014a). High sulfate concentrations, exceeding threefold the maximum allowance level set for drinking water in the European Union (Directive 98/83/EC 1998), were noted at all sampling stations in August. Periodically increased levels of this parameter are observed in surface waters of this region and can be, at least partially, explained by high sulfate concentration in soil (Choban and Winkler 2010; Peryt et al. 2012). In general, the studied waters were eutrophic as indicated by inorganic nitrogen and phosphate concentrations. This was also evident from phytoplankton analyses—cyanobacteria were a dominant fraction in all the studied samples in both August and September, with abundance ranging from 78% to as much as 98% depending on period and studied site (Fig. 2). A share of each identified cyanobacterial species in the total phytoplankton and cyanobacteria community is given in Table 2. The dominant taxa at KHPP and RS sites included *Pseudanabaena* sp. and *Planktothrix agardhii* (Fig. 3c), whereas *P*. *agardhii* and *Planktolyngbya limnetica* dominated at the KAPP site. *M*. *aeruginosa* was identified only at the KAPP site and only in September with a 17% share in total phytoplankton. *C*. *raciborskii* (Fig. 3a) and *Sphaerospermopsis aphanizomenoides* (Fig. 3d) were identified at the KHPP and RS sites with their share in total phytoplankton reaching a maximum of 8.5 and 6.3%, respectively. *A*. *gracile* was identified at all studied sites but at very low abundances (Table 2).

**Table 1:** Physicochemical parameters of water at sampling sites

Parameter	KHPP		RS		KAPP	
	August	September	August	September	August	September
Temperature [°C]	23.3	15.1	25.2	14.9	28.1	17.7
рН	7.45 ± 0.06	8.00 ± 0.05	7.52 ± 0.07	7.77 ± 0.05	8.10 ± 0.05	7.80 ± 0.05
NH_4_^+^ [mg/L]	0.23 ± 0.03	0.85 ± 0.05	0.26 ± 0.03	1.25 ± 0.25	0.33 ± 0.02	1.55 ± 0.75
NO_2_^−^ [mg/L]	0.01 ± 0.005	0.11 ± 0.01	0.01 ± 0.005	0.11 ± 0.005	0.01 ± 0.005	0.01 ± 0.01
NO_3_^−^ [mg/L]	0.49 ± 0.05	1.45 ± 0.19	0.53 ± 0.06	2.10 ± 0.09	0.20 ± 0.02	0.03 ± 0.05
Cl^−^ [mg/L]	40.32 ± 1.50	28.36 ± 1.75	39.10 ± 1.50	29.00 ± 1.65	49.63 ± 2.15	56.72 ± 2.50
SO_4_^2−^ [mg/L]	796.0 ± 76.2	220.0 ± 15.6	740.0 ± 57.7	240.0 ± 20.0	744.5 ± 33.9	150.5 ± 12.9
PO_4_^2+^ [mg/L]	0.41 ± 0.005	0.14 ± 0.02	0.38 ± 0.005	0.13 ± 0.03	0.386 ± 0.002	0.16 ± 0.007
Phenol [μg/L]	0.20 ± 0.02	0.23 ± 0.02	0.18 ± 0.02	0.22 ± 0.05	1.00 ± 0.04	0.13 ± 0.04
Dry residue [mg/L]	748 ± 31	825	702 ± 35	750	916 ± 43	725
Total Ca [mg/L]	61.2 ± 1.9	76.1 ± 1.2	65.7 ± 1.3	78.4 ± 1.8	68.3 ± 2.6	65.9 ± 1.4
Total Fe [mg/L]	0.07 ± 0.01	0.04 ± 0.005	0.25 ± 0.1	0.03 ± 0.002	0.10 ± 0.001	0.02 ± 0.001
Total K [mg/L]	5.52 ± 0.6	5.88 ± 0.2	4.70 ± 0.1	5.19 ± 0.3	7.26 ± 0.3	6.42 ± 0.2
Total Mg [mg/L]	9.25 ± 0.5	8.24 ± 0.1	8.6 ± 0.1	8.53 ± 0.1	8.52 ± 0.3	8.1 ± 0.2
Total Na [mg/L]	10.51 ± 0.3	10.2 ± 0.1	10.28 ± 0.1	10.0 ± 0.2	39.52 ± 1.5	36.8 ± 0.2
Total Mn [mg/L]	0.06 ± 0.3	0.05 ± 0.002	0.18 ± 0.08	0.04 ± 0.01	0.05 ± 0.03	0.01 ± 0.01
Total Al [mg/L]	0.55 ± 0.2	0.15 ± 0.06	0.044 ± 0.03	0.019 ± 0.01	0.01 ± 0.001	0.014 ± 0.001
Total Cd [mg/L]	< LOD	0.002 ± 0.001	0.001 ± 0.001	< LOD	< LOD	< LOD
Total Co [mg/L]	0.004 ± 0.001	0.004 ± 0.001	0.004 ± 0.001	0.005 ± 0.001	0.004 ± 0.001	0.004 ± 0.001
Total Cr [mg/L]	0.004 ± 0.001	0.004 ± 0.001	0.004 ± 0.001	0.005 ± 0.001	0.004 ± 0.001	0.004 ± 0.001
Total Cu [mg/L]	< LOD	0.005 ± 0.001	< LOD	0.015 ± 0.001	0.005 ± 0.001	0.032 ± 0.001
Total Hg [mg/L]	< LOD	< LOD	< LOD	< LOD	< LOD	< LOD
Total Ni [mg/L]	0.007 ± 0.001	0.007 ± 0.001	0.004 ± 0.001	< LOD	0.012 ± 0.001	0.008 ± 0.001
Total Pb [mg/L]	0.011	0.018	0.045	0.075	0.024	0.042
Total Zn [mg/L]	< LOD	< LOD	< LOD	< LOD	< LOD	0.005 ± 0.001

*< LOD* below limit of detection

**Table 2:** The cyanobacteria species identified at studied sites

	Share in total phytoplankton (%)			Share in total cyanobacteria (%)		
	August/September					
	KHPP	RS	KAPP	KHPP	RS	KAPP
*Anabaenopsis cunningtonnii*	0.0/0.1	0.3/0.8	0.0/0.0	0.0/0.2	0.3/0.9	0.0/0.0
*Anabaenopsis elenkinii*	0.0/0.0	0.1/0.0	0.0/0.0	0.0/0.0	0.1/0.0	0.0/0.0
*Aphanizomenon gracile*	1.5/6.9	1.5/3.9	0.3/0.0	1.5/7.4	1.6/4.2	0.4/0.0
*Cuspidothrix issatschenkoi*	0.3/0.0	0.3/0.0	0.0/0.0	0.3/0.0	0.3/0.0	0.0/0.0
*Cylindrospermopsis raciborskii*	3.9/8.5	2.7/1.1	0.0/0.0	4.0/9.2	2.8/1.1	0.0/0.0
*Dolichospermum* sp.	0.3/2.3	0.0/2.4	0.0/0.0	0.3/2.5	0.0/2.5	0.0/0.0
*Dolichospermum flos-aquae*	1.3/0.5	0.7/7.1	0.0/0.0	1.4/0.6	0.7/7.5	0.0/0.0
*Limnothrix redeckei*	0.7/0.0	0.1/0.3	0.0/0.0	0.7/0.0	0.7/0.3	0.0/0.0
*Merismopedia tenuissima*	0.1/0.0	0.0/0.6	0.0/0.0	0.2/0.0	0.0/0.6	0.0/0.0
*Microcystis aeruginosa*	0.0/0.0	0.4/0.0	0.0/17.0	0.0/0.0	0.4/0.0	0.0/18.4
*Planktothrix agardhii*	25.9/38.8	72.3/34.1	54.7/75.5	26.8/42.0	73.8/36.3	69.6/81.6
*Planktolyngbya limnetica*	8.2/0.0	1.4/0.0	18.2/0.0	8.4/0.0	1.4/0.0	23.1/0.0
*Pseudanabaena* sp.	49.0/35.3	12.0/43.8	5.4/0.0	50.6/38.2	12.2/46.6	6.8/0.0
*Sphaerospermopsis aphanizomenoides*	5.6/0.0	6.2/0.0	0.0/0.0	5.8/0.0	6.3/0.0	0.0/0.0

This report highlights the presence of number of potent toxin-producing cyanobacteria in man-made water reservoirs in Ukraine. Although species such as *C*. *raciborskii* and *M*. *aeruginosa* have been reported earlier in Ukraine (Novoselova and Protasov 2016), the data on their potential toxicity is scarce and focused only on MCs. As provided by Belykh et al. (2013), screening of *mcy* genes in the freshwater of Kiev regions yielded positive results in the majority of studied samples, although exact producers were not identified. Previous phytoplankton screening conducted in 2005 in the Kasperivtsi Hydrothermal Power Plant reservoir and outflowing River Seret did not identify *C*. *raciborskii*, *P*. *agardhii*, or *A*. *gracile* (Shcherbak and Bondarenko 2005). Therefore, the findings of the present study indicate the potential expansion of these cyanobacteria in this region and highlight that their further dispersion through a river system is plausible.

Our study identified two main potent producers of MCs: *M*. *aeruginosa* and *P*. *agardhii*, but even though the latter reaches a relatively high abundance, the dissolved and particulate content of all three studied MC analogues was found below detection limits. However, the occurrence of non-MC-producing strains of both species is a common phenomenon (Yéprémian et al. 2007; Park et al. 2018), and previous studies have indicated that the temporal and spatial distribution of toxic and non-toxic genotypes can be attributed to a number of factors including resource competition (Briand et al. 2008; Lei et al. 2015; Suominen et al. 2017).

Potential ANA-A-producing cyanobacteria identified in this study included *Cuspidothrix issatschenkoi* (Usačev) Rajaniemi et al. (Ballot et al. 2010) and *Dolichospermum flos-aquae* (Lyngbye) Wacklin, Hoffmann et Komárek (Osswald et al. 2009), although this neurotoxin was not detected. Moreover, no dissolved or particulate CYN was found in the studied samples, excluding its production by the identified strain of *A*. *gracile* (Fig. 1b), a species previously demonstrated as a major CYN producer in Poland (Kokociński et al. 2013). Our findings also support a preliminary view that Ukrainian strains of *C*. *raciborskii* are unable to produce CYN. The abundance of this species in the studied waters was relatively low, in line with previous observations in Polish lakes where *C*. *raciborskii* biomass, even during cyanobacterial blooms, was usually not high, and maximally accounted for 40% of total phytoplankton biomass (Kokocinski et al. 2017). However, there was a single reported case when *C*. *raciborskii* dominated the phytoplankton of a shallow reservoir in Poland after a particularly hot and sunny early summer (Budzyńska and Gołdyn 2017). Moreover, long-term phytoplankton analysis in Poland showed the ability of *C*. *raciborskii* to outcompete a native bloom-forming cyanobacterial species *Planktothrix agardhii* (Kokociński et al. 2010). It was also experimentally evidenced that the Polish strain of *C*. *raciborskii* can outcompete *M*. *aeruginosa* even at relatively low initial biomass (Rzymski et al. 2014b). Therefore, the dynamic of *C*. *raciborskii* occurrence in Ukraine surface waters requires further monitoring.

**Fig. 4:**
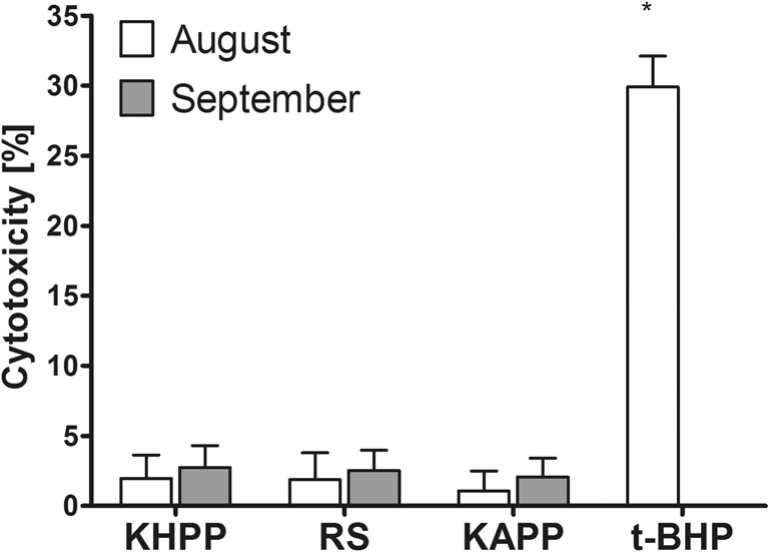
The cytotoxicity of filtered water samples in human platelet-rich plasma assessed by means of lactate dehydrogenase leakage assay and compared to that exerted by tert-butyl hydroperoxide (t-BHP; positive control). Asterisk indicates statistically significant difference with control (*p* < 0.05; Student *t* test)

Additionally, the collected waters were screened for their toxic properties in human PRP in vitro. The employed model is convenient as it allows a study of reactions in the presence of extracellular components while platelets were previously reported to respond to toxic compounds during short-time exposure and release LDH during their lysis (Kim et al. 2009). It was found that the collected waters did not display a significant in vitro toxicity in human platelets (< 3% cytotoxicity) as evidenced using an LDH assay (Fig. 4). This rather excludes the presence of significant concentrations of some other toxic compounds in the collected water.

In summary, this study explored the presence of cyanobacteria in the water reservoirs of power plants in Ukraine demonstrating that this group dominated phytoplankton during summer. Although potent producers of CYN, ANA-A, and MCs were identified, the intra- and extracellular presence of these cyanotoxins was not confirmed. Further toxicological and ecological studies, including molecular investigations on isolated strains, are required to evaluate the presence of potent cyanotoxin producers and associated health threats in this geographical region.
